# Supplementary material for: Relaxed 3D genome conformation facilitates the pluripotent to totipotent-like state transition in embryonic stem cells
Source: Nucleic Acids Res. 2021 Nov 17;49(21):12167–77. doi: 10.1093/nar/gkab1069 (PMC8643704; doi:10.1093/nar/gkab1069)
Supplement: gkab1069_Supplemental_Files [file gkab1069_supplemental_files.zip › Supplementary Figures2021-11-01.pdf]

Supplementary Information

Relaxed 3D genome conformation facilitates the pluripotent to totipotent-like state transition in embryonic stem cells

Supplementary Table S1. Sequencing summary for Hi-C, RNA-seq, ATAC-seq, ChIP-seq, and CUT&Tag libraries generated in this study.

Supplementary Table S2. Sequences for real-time qPCR primers and siRNAs used in this study.

Supplementary Figures S1-S5

Figure S1

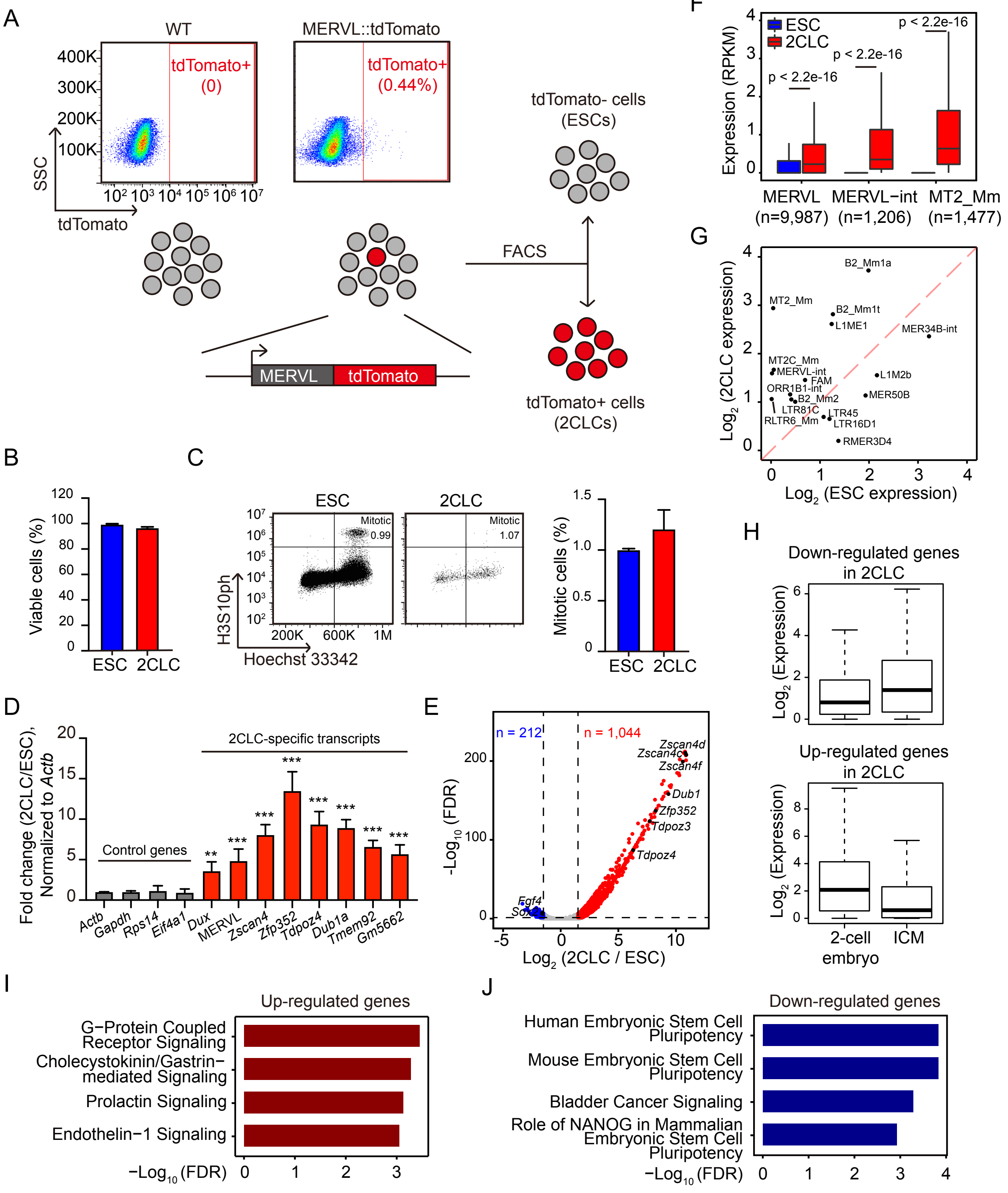

A
WT
MERVL::tdTomato
tdTomato+
(0)
tdTomato+
(0.44%)
SSC
tdTomato
FACS
tdTomato- cells
(ESCs)
tdTomato+ cells
(2CLCs)
MERVL
tdTomato
B
Viable cells (%)
ESC
2CLC
C
ESC
2CLC
Mitotic
0.99
Mitotic
1.07
H3S10ph
Hoechst 33342
Mitotic cells (%)
D
Fold change (2CLC/ESC), Normalized to Actb
Control genes
2CLC-specific transcripts
Actb
Gapdh
Rps14
Eif4a1
Dux
MERVL
Zscan4
Zfp352
Tdpoz4
Dub1a
Tmem92
Gm5662
E
n = 212
n = 1,044
Zscan4d
Zscan4c
Zscan4f
Dub1
Zfp352
Tdpoz3
Tdpoz4
Fgf4
Sox2
-Log10 (FDR)
Log2 (2CLC / ESC)
F
ESC
2CLC
p < 2.2e-16
Expression (RPKM)
MERVL
(n=9,987)
MERVL−int
(n=1,206)
MT2_Mm
(n=1,477)
G
B2_Mm1a
MT2_Mm
B2_Mm1t
L1ME1
MER34B-int
MT2C_Mm
MERVL-int
FAM
ORR1B1-int
L1M2b
B2_Mm2
MER50B
LTR81C
LTR45
RLTR6_Mm
LTR16D1
RMER3D4
Log2 (2CLC expression)
Log2 (ESC expression)
H
Down-regulated genes
in 2CLC
Up-regulated genes
in 2CLC
Log2 (Expression)
2-cell
embryo
ICM
I
Up-regulated genes
G−Protein Coupled
Receptor Signaling
Cholecystokinin/Gastrin−
mediated Signaling
Prolactin Signaling
Endothelin−1 Signaling
−Log10 (FDR)
J
Down-regulated genes
Human Embryonic Stem Cell
Pluripotency
Mouse Embryonic Stem Cell
Pluripotency
Bladder Cancer Signaling
Role of NANOG in Mammalian
Embryonic Stem Cell
Pluripotency
−Log10 (FDR)

Supplementary Figure S1. RNA-seq validates the fidelity of the purified 2CLCs. (**A**) Schematic representation of 2CLC purification. (**B**) Bar plot showing the viability of ESCs and 2CLCs as measured by 7-AAD/Annexin V staining followed by flow cytometry analysis. (**C**) Representative FACS plot (left) and a bar plot (right) showing the percentages of mitotic cells in ESCs and 2CLCs. (**D**) Relative expression levels of 2C-specific transcripts in 2CLCs. RT-qPCR results were normalized to *Actin* and are presented as mean ± SD, $^{**}p < 0.01$, $^{***}p < 0.001$ (multiple *t* tests). (**E**) Scatter plot comparing the gene expression between ESCs and 2CLCs. Significantly up-regulated and down-regulated genes (FDR < 0.05, |log2FC| > 1.5) are highlighted in red and blue, respectively. (**F**) Box plot showing expression of MERVL, MERVL-int, and MT2_Mm in ESCs and 2CLCs. (**G**) Scatter plot showing expression levels of indicated repeats in ESCs and 2CLCs. (**H**) Box plots showing the expression levels of down-regulated (upper panel) and up-regulated genes (lower panel) detected in 2CLC in 2-cell embryos and ICM. (**I-J**) Ingenuity pathway analysis of up-regulated genes (I) and down-regulated genes (J) in 2CLCs.

Figure S2

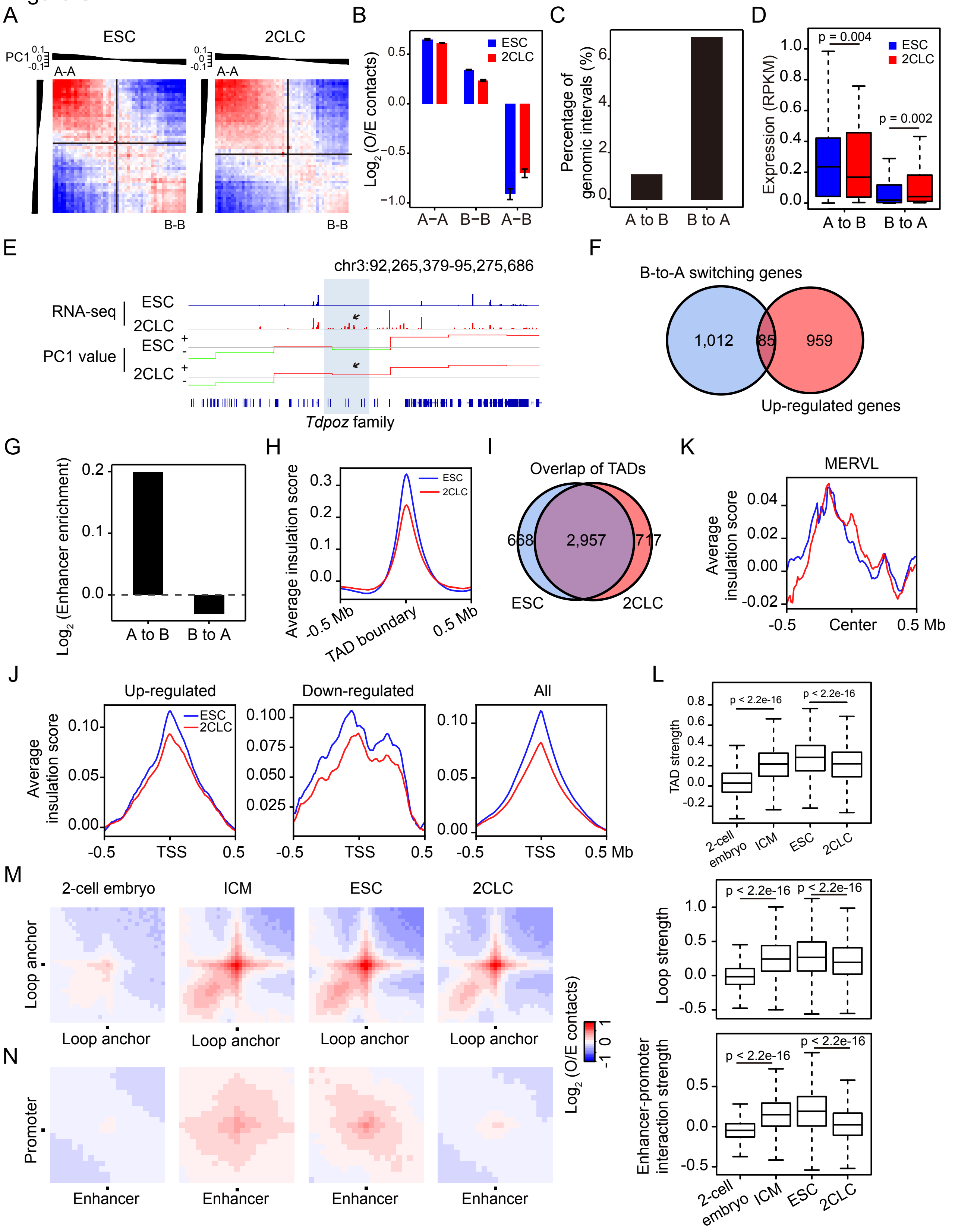

A
ESC
2CLC
PC1
A-A
B-B
B
Log2 (O/E contacts)
ESC
2CLC
A–A
B–B
A–B
C
Percentage of genomic intervals (%)
A to B
B to A
D
Expression (RPKM)
p = 0.004
p = 0.002
ESC
2CLC
A to B
B to A
E
chr3:92,265,379-95,275,686
RNA-seq
ESC
2CLC
PC1 value
ESC
2CLC
Tdpoz family
F
B-to-A switching genes
1,012
85
959
Up-regulated genes
G
Log2 (Enhancer enrichment)
A to B
B to A
H
Average insulation score
ESC
2CLC
-0.5 Mb
TAD boundary
0.5 Mb
I
Overlap of TADs
668
2,957
717
ESC
2CLC
K
MERVL
Average insulation score
-0.5
Center
0.5 Mb
J
Up-regulated
Down-regulated
All
Average insulation score
ESC
2CLC
-0.5
TSS
0.5
0.5 Mb
L
TAD strength
p < 2.2e-16
p < 2.2e-16
2-cell embryo
ICM
ESC
2CLC
M
2-cell embryo
ICM
ESC
2CLC
Loop anchor
Loop strength
N
Promoter
Enhancer
Log2 (O/E contacts)
Enhancer-promoter interaction strength

Supplementary Figure S2. 3D genome conformation in ESCs and 2CLCs. (**A**) Hi-C contact maps between pairs of 500-kb loci arranged by their PC1 values (shown on top and left) across chromosome 1. (**B**) Bar plot showing O/E contact strength between domains from the same ("A" versus "A" or "B" versus "B") and different ("A" versus "B") compartment in ESC and 2CLC. Values represent means of two biological replicates with ends of error bars corresponding to individual data points. (**C**) Bar plot showing the percentage of genomic intervals which display A/B compartment switch during ESC to 2CLC transition. (**D**) Box plot showing the transcriptional level of regions display A/B compartment switch. (**E**) Genome browser view showing RNA-seq signal and PC1 values at *Tdpoz* locus in ESC and 2CLC. Green = B compartment; red = A compartment; Blue box = *Tdpoz* locus. (**F**) Venn diagram showing the overlap of B-to-A switching genes and up-regulated genes in 2CLC. (**G**) Bar plot showing log2 ratio of ESC enhancers to control regions located in A/B compartment switches regions. Equal number of random regions were used as control. (**H**) Average insulation score in a 0.5 Mb region centered on TAD boundaries. (**I**) Venn diagram showing the overlap of TADs identified in ESC and 2CLC. (**J-K**) Average insulation score around TSS of indicated genes (J) and MERVL repeats (K) in ESCs and 2CLCs. (**L**) Box plot comparing TAD strengths in 2C embryos, ICM, ESCs, and 2CLCs. (**M**) Aggregate Hi-C contact maps between pairs of loop anchors in 2C embryos, ICM, ESCs, and 2CLCs (left). Box plot showing loop strength in 2Cell, ICM, ESC, and 2CLC (right). (**N**) Aggregate Hi-C contact maps between ESC enhancer-promoter pairs in 2C embryo, ICM, ESCs, and 2CLCs (left). Box plot showing loop strength in 2C embryos, ICM, ESCs, and 2CLCs (right).

Figure S3

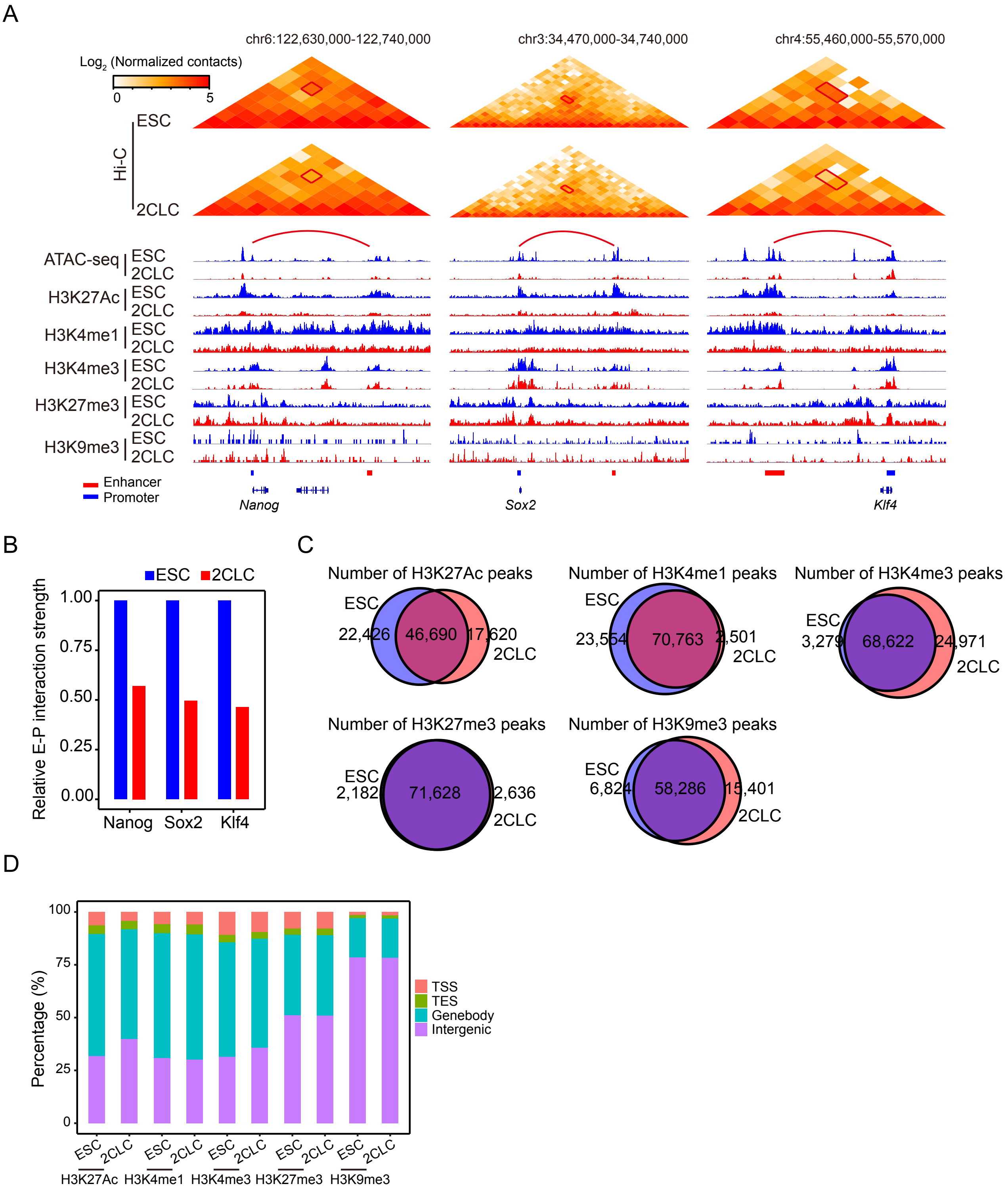
A
chr6:122,630,000-122,740,000
chr3:34,470,000-34,740,000
chr4:55,460,000-55,570,000
Log2 (Normalized contacts)
0
5
Hi-C
ESC
2CLC
ATAC-seq
H3K27Ac
H3K4me1
H3K4me3
H3K27me3
H3K9me3
Enhancer
Promoter
Nanog
Sox2
Klf4
B
ESC
2CLC
Relative E-P interaction strength
1.00
0.75
0.50
0.25
0.00
Nanog
Sox2
Klf4
C
Number of H3K27Ac peaks
22,426
46,690
17,620
Number of H3K4me1 peaks
23,554
70,763
2,501
Number of H3K4me3 peaks
3,279
68,622
24,971
Number of H3K27me3 peaks
2,182
71,628
2,636
Number of H3K9me3 peaks
6,824
58,286
15,401
D
Percentage (%)
100
75
50
25
0
TSS
TES
Genebody
Intergenic
H3K27Ac
H3K4me1
H3K4me3
H3K27me3
H3K9me3

Supplementary Figure S3. Loss of enhancer-promoter interactions of pluripotent genes in 2CLCs. (**A**) Hi-C contact maps near *Nanog*, *Sox2*, and *Klf4* (top). Genome browser tracks of ATAC, H3K27Ac, H3K4me1, H3K4me3, H3K27me3, and H3K9me3 ChIP-seq signals in the corresponding region (bottom). An arc representing an enhancer-promoter interaction. The signal in the outlined pixels was used to quantify the change in enhancer-promoter interaction strength between ESC and 2CLC. (**B**) Bar plot showing the relative enhancer-promoter interaction strength in ESC and 2CLC at the *Nanog*, *Sox*, and *Klf4* locus, which are outlined in (A). E-P, enhancer-promoter. (**C**) Venn diagram showing the overlap of H3K27Ac, H3K4me1, H3K4me3, H3K27me3, and H3K9me3 ChIP-seq peaks identified in ESCs and 2CLCs. (**D**) Genomic distributions of H3K27Ac, H3K4me1, H3K4me3, H3K27me3, and H3K9me3 ChIP-seq peaks in ESCs and 2CLCs.

Figure S4

A

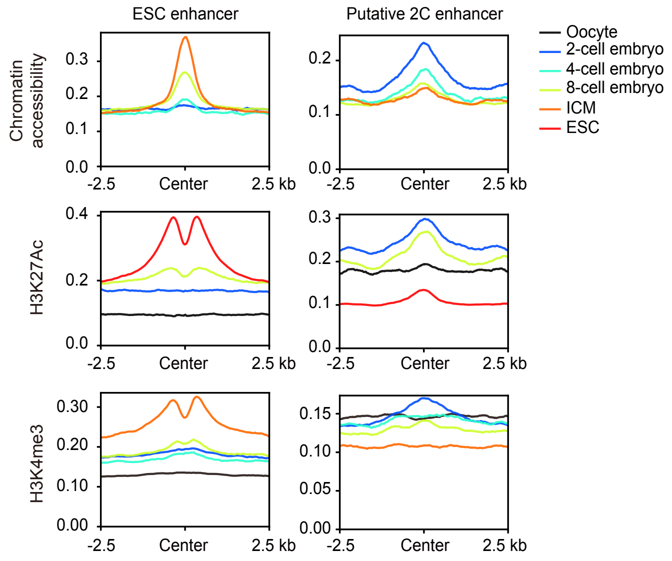
ESC enhancer
Putative 2C enhancer
Oocyte
2-cell embryo
4-cell embryo
8-cell embryo
ICM
ESC
Chromatin accessibility
H3K27Ac
H3K4me3
-2.5
Center
2.5 kb

B

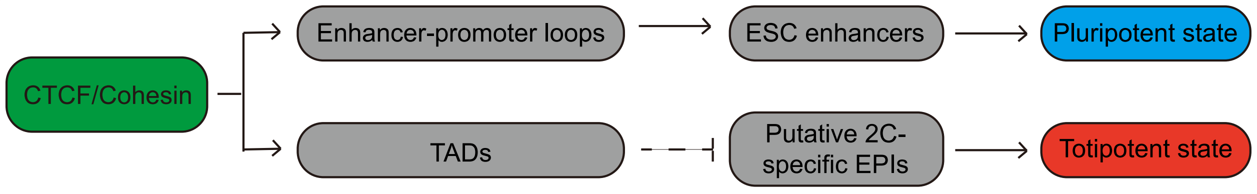
CTCF/Cohesin
Enhancer-promoter loops
ESC enhancers
Pluripotent state
TADs
Putative 2C-specific EPIs
Totipotent state

Supplementary Figure S4. Shifts between ESC enhancers and putative 2C enhancers. (**A**) Average ATAC, H3K27Ac, and H3K4me3 signals in 2.5 kb region centered on ESC enhancers and 2C enhancers in oocyte, 2-cell, 4-cell, 8-cell, ICM embryos, and ESCs. (**B**) A model showing that disruption of TADs and enhancer-promoter loops facilitates the ESC to 2CLC transition.

Figure S5

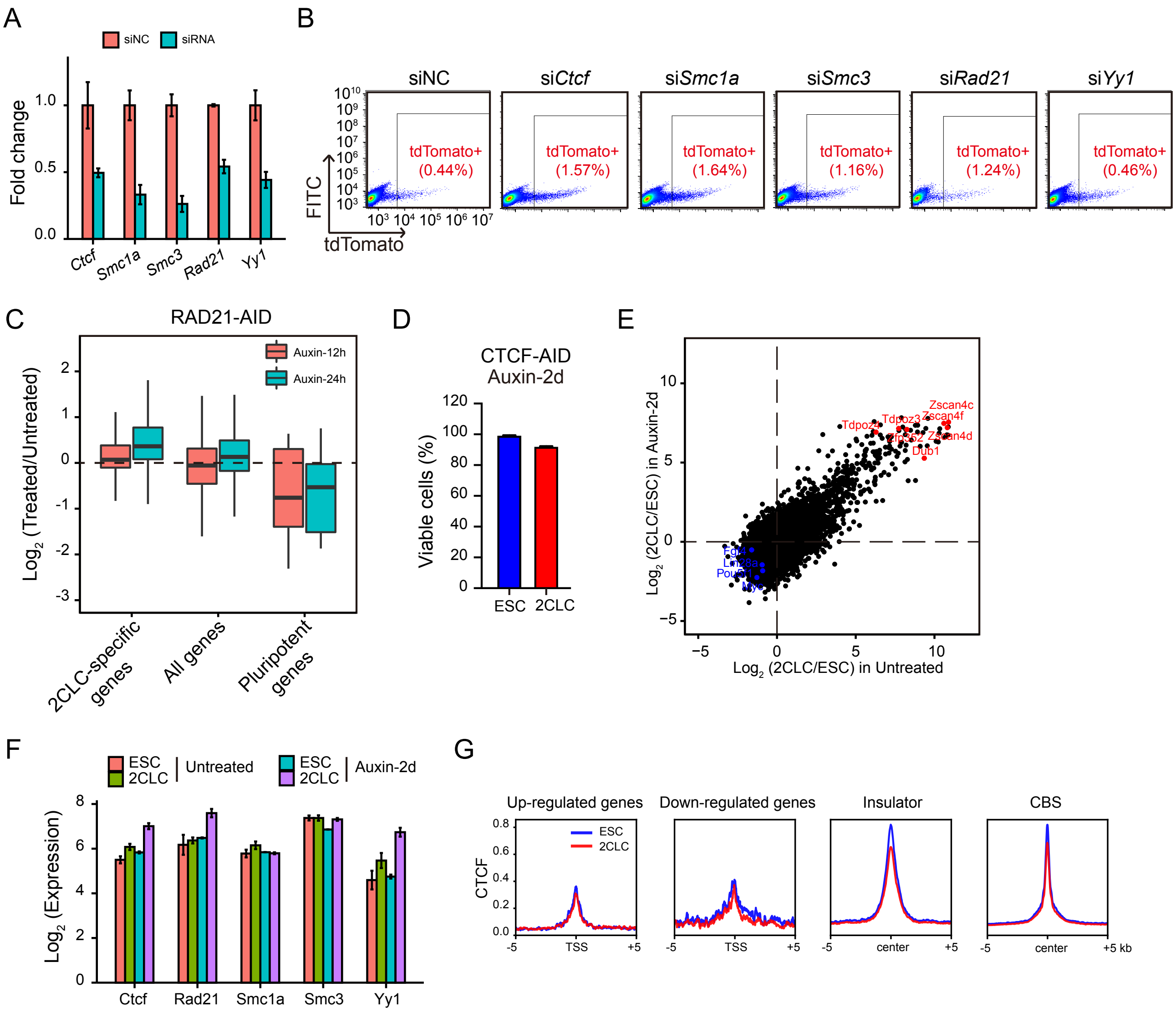

A
siNC
siRNA
Fold change
1.0
0.5
0.0
Ctcf
Smc1a
Smc3
Rad21
Yy1
B
siNC
siCtcf
siSmc1a
siSmc3
siRad21
siYy1
tdTomato+ (0.44%)
tdTomato+ (1.57%)
tdTomato+ (1.64%)
tdTomato+ (1.16%)
tdTomato+ (1.24%)
tdTomato+ (0.46%)
FITC
tdTomato
C
RAD21-AID
Auxin-12h
Auxin-24h
Log2 (Treated/Untreated)
2CLC-specific genes
All genes
Pluripotent genes
D
CTCF-AID
Auxin-2d
Viable cells (%)
ESC
2CLC
E
Log2 (2CLC/ESC) in Auxin-2d
Log2 (2CLC/ESC) in Untreated
Zscan4c
Zscan4f
Zscan4d
Tdpoz4
Tdpoz3
Dub1
F
ESC
2CLC
Untreated
Auxin-2d
Log2 (Expression)
Ctcf
Rad21
Smc1a
Smc3
Yy1
G
Up-regulated genes
Down-regulated genes
Insulator
CBS
CTCF
ESC
2CLC
TSS
center
+5 kb

Supplementary Figure S5. Depletion of CTCF or cohesin facilitates ESC to 2CLC transition. (**A**) Bar plot showing knockdown efficiencies of indicated siRNAs. (**B**) Representative FACS plots showing percentages of 2CLCs upon knockdown of *Ctcf*, *Smc1a*, *Smc3*, *Rad21*, or *Yy1*. (**C**) Box plot showing log2 fold change of indicated groups of genes upon acute depletion of the RAD21 protein in ESCs by the auxin-inducible degron (AID). Analyses were performed using a published RNA-seq dataset (12). (**D**) Bar plot showing the viability of ESCs and 2CLCs in CTCF depleted cells (2 days after auxin treatment) as measured by 7-AAD/Annexin V staining followed by flow cytometry analysis. (**E**) Scatter plot comparing log2 fold changes of gene expression in 2CLCs in the presence (Untreated) and absence of CTCF (Auxin-2d). (**F**) Bar plot showing expression levels of architecture genes in ESCs and 2CLCs either in the presence (Untreated) or in the absence of CTCF (Auxin-2d) as revealed by RNA-seq analysis of the sorted cells. (**G**) Average CTCF binding signals around indicated loci in ESCs and 2CLCs. CBS, CTCF binding sites identified in ESCs.
